# Nucleoredoxin Plays a Key Role in the Maintenance of Retinal Pigmented Epithelium Differentiation

**DOI:** 10.3390/antiox11061106

**Published:** 2022-06-01

**Authors:** Mariana I. Holubiec, Juan I. Romero, Claudia Urbainsky, Manuela Gellert, Pablo Galeano, Francisco Capani, Christopher Horst Lillig, Eva-Maria Hanschmann

**Affiliations:** 1 Facultad de Medicina, Instituto de Investigaciones Cardiológicas “Prof. Dr. Alberto C. Taquini” (ININCA), Universidad de Buenos Aires (UBA-CONICET), Buenos Aires 1122, Argentina; fcapani@fmed.uba.ar; 2 Instituto de Investigación en Biomedicina de Buenos Aires, Partner Institute of the MaxPlank Society (IBioBA-CONICET-MPSP), Buenos Aires 2390, Argentina; 3 Instituto de Investigaciones Bioquímicas de Buenos Aires (IIBBA-CONICET), Fundación Instituto Leloir, Buenos Aires 1405, Argentina; jromero@leloir.org.ar (J.I.R.); pgaleano@leloir.org.ar (P.G.); 4 Institute for Medical Biochemistry and Molecular Biology, University Medicine Greifswald, University of Greifswald, 17489 Greifswald, Germany; c_urbainsky@web.de (C.U.); gellertm@uni-greifswald.de (M.G.); horst@lillig.de (C.H.L.); 5 Facultad de Medicina, Universidad Católica Argentina (UCA), Buenos Aires 1600, Argentina; 6 Department of Neurology, Medical Faculty, Heinrich-Heine University, 40225 Düsseldorf, Germany

**Keywords:** nucleoredoxin, hypoxia-ischemia, reoxygenation, retina, RPE, differentiation, morphology, neurons, glia, redox regulation

## Abstract

Nucleoredoxin (Nrx) belongs to the Thioredoxin protein family and functions in redox-mediated signal transduction. It contains the dithiol active site motif Cys-Pro-Pro-Cys and interacts and regulates different proteins in distinct cellular pathways. Nrx was shown to be catalytically active in the insulin assay and recent findings indicate that Nrx functions, in fact, as oxidase. Here, we have analyzed Nrx in the mammalian retina exposed to (perinatal) hypoxia-ischemia/reoxygenation, combining ex vivo and in vitro models. Our data show that Nrx regulates cell differentiation, which is important to (i) increase the number of glial cells and (ii) replenish neurons that are lost following the hypoxic insult. Nrx is essential to maintain cell morphology. These regulatory changes are related to VEGF but do not seem to be linked to the Wnt/β-catenin pathway, which is not affected by Nrx knock-down. In conclusion, our results strongly suggest that hypoxia-ischemia could lead to alterations in the organization of the retina, related to changes in RPE cell differentiation. Nrx may play an essential role in the maintenance of the RPE cell differentiation state via the regulation of VEGF release.

## 1. Introduction

Nucleoredoxin (Nrx) is a member of the Thioredoxin (Trx) family of proteins that catalyze redox-reactions and mediate redox signaling [1]. Nrx was first described in mice as a redox protein with the dithiol active site motif Cys-Pro-Pro-Cys that shares high similarities with the classical Trx active site motif Cys-Gly-Pro-Cys [1]. Nrx was thought to be exclusively expressed in the nucleus and was therefore named Nucleoredoxin [2,3]. However, it was later shown to be localized mainly in the cytosol [2]. Nrx has oxidoreductase activity [1,2]. Interestingly, maize Nrx was shown to have similar kinetics as E. coli Trx1 in the insulin reduction assay [3]. Besides the dithiol Trx-like domain, Nrx contains two monothiol Trx-like domains and a C-terminal PDI-like domain resembling the substrate recognition region of protein disulfide isomerases (PDIs) [2]. Previous reports demonstrated that Nrx is involved in the regulation of the Wnt/β-catenin pathway. Nrx interacts with and regulates Dishevelled (Dvl), an important downstream mediator in the Wnt signaling pathway [4]. Moreover, Nrx is essential to maintain Dvl levels, preventing its degradation and retaining a pool of inactive Dvl protein [4]. Previous works, using redox probe carboxy-H_2_DCFDA and MitoTracker red CMXRos, as well as H_2_O_2_ treatments, showed that an increase in H_2_O_2_ produced in the mitochondria promotes the dissociation of Nrx and Dvl [5]. These molecular changes activate the Wnt/β-catenin pathway that regulates neuronal differentiation, early development and stem cell maintenance [4,5,6,7]. Kneeshaw et al. [8] found that Nrx interacts with several enzymes that play a role in the cellular H_2_O_2_-scavenging pathway. Studies in plant cells have shown that H_2_O_2_ is detoxified by catalases and ascorbate peroxidase (APX) enzymes, all of which were found to interact with Nrx [8]. Our recent data support the hypothesis that Nrx functions as an oxidase, rather than a reductase. In fact, knock-down of Nrx in SH-SY5Y cells and redox proteomic analysis revealed that the proteome was significantly more reduced in the absence of Nrx [9].

Several molecular and biochemical changes occur during neonatal hypoxia-ischemia (H-I), including redox alterations and neuroinflammation [10]. A higher production of reactive species, particularly nitric oxide (NO) and peroxynitrite, was shown to affect the central nervous system (CNS) [11,12]. The decrease in O_2_ in astrocytes leads to a diminution in the production of ATP that generates changes in membrane polarization due to a reduced activity of the Na/K ATPase pump. Large amounts of glutamate (Glu) produced in neurons lead to postsynaptic membrane polarization changes due to the activation of different Glu-dependent Ca^2+^ channels. This, in turn, leads to an increase in the intracellular Ca^2+^ levels that induce a higher expression of neuronal nitric oxide synthase (nNOS) and thus induce NO synthesis. Upon reoxygenation, antioxidant enzymes, such as superoxide dismutase, whose expression is already low in the CNS, have been oxidized by reactive species produced during the hypoxic insult. Thus, the system is not capable of coping with the fresh influx of O_2_ that generates new reactive oxygen species (ROS), including NO, superoxide anion (O_2_•^−^) and hydrogen peroxide (H_2_O_2_) [11,12,13,14,15,16]. Hypoxia, followed by reperfusion, produces several cellular and molecular changes including an inflammatory cascade, damage to macromolecules, such as proteins involved in the respiratory chain, and changes in the expression of redox related proteins [17,18,19,20]. The retina is a special tissue that undergoes high levels of stress due to its exposure to elevated oxygen levels and UV light. The neural retina is exposed to both light and elevated oxygen levels under physiological conditions and produces a great number of ROS, such as singlet oxygen, O_2_•^−^ and H_2_O_2_ [21,22].

Retinal pigmented epithelium (RPE) cells play an important role in the organization of the spatial structure of the retina. The interactions of RPE cells, a mosaic of postmitotic polarized cuboidal cells and photoreceptors are essential for differentiation into cells belonging to different retinal layers and maintaining the visual function [23,24]. In response to various environmental stimuli, such as inflammation, injury and aging, RPE cells rapidly proliferate. While RPE cells are able to quickly re-establish a continuous monolayer in mild injuries, they cannot create a new functional retina-RPE complex following more severe insults [24]. RPE cells are located near the choroidal capillaries, making them particularly susceptible to ischemia or hypoxia [25]. In fact, H-I and subsequent reperfusion generate an increment in reactive species. This increase causes neuronal loss and can ultimately lead to blindness, as it occurs in retinopathy of prematurity (ROP) [26,27]. Exposure to H-I/reperfusion induces the expression of hypoxia inducible factor-1α (Hif1α) and its target genes, e.g., vascular endothelial growth factor (VEGF). As angiogenic factor VEGF contributes to the thickening of the inner retina layers [28]. VEGF is known to be involved in vascular alterations associated with leading causes of blindness, i.e., age-related macular degeneration, diabetic retinopathy and retinal vein occlusions [29]. The inappropriate retinal neovascularization as a result of endothelial cell survival during tissue hypoxia, e.g., in diabetic retinopathy, may be the result of a VEGF dependent activation of the JAK/STAT (Janus kinase/signal transduction and activator of transcription) pathway [30]. VEGF synthesized by differentiated RPE cells is necessary for photoreceptor differentiation. The lack of RPE cell derived VEGF causes atrophy on the choriocapillaris and thereby disrupts the photoreceptor differentiation, leading to photoreceptor apoptosis [31].

Nrx regulates cell differentiation, a process necessary to (i) increase the number of glial cells and (ii) replenish the number of neurons that are lost following a hypoxic insult. In the present study, we aimed to determine the role of Nrx in the mammalian retina exposed to H-I using an ex vivo animal model as well as an in vitro cell model of RPE. We were able to observe changes in Nrx regulation due to H-I in the retina and particularly in the RPE. Furthermore, using a stable RPE cell line, we determined that Nrx plays a key role in cell morphology and in the differential expression of differentiation markers in these cells, leading us to believe that differentiation of RPE cells is related to Nrx expression.

## 2. Materials and Methods

### 2.1. Chemicals and Reagents

Chemicals and enzymes used in the present work were of analytical grade or better. Reagents were purchased from Sigma-Aldrich (Munich, Germany), unless otherwise stated. SDS-PAGE was run using 4–20% Mini PROTEAN TGX precasted stain-free gels (Biorad, Munich, Germany) according to the manufacturer’s instructions. For protein transfer the Turbo RTA Transfer Kit with PVDF membranes (Biorad, Munich, Germany) was used.

### 2.2. Ethical Statement

All animal procedures were approved by the “Comité Institucional para el Cuidado y Uso de Animales de Laboratorio” (CICUAL, Resolución N°2238/2010) of the School of Medicine of the University of Buenos Aires. In addition, all experimental procedures were carried out under strict adherence to the European Directive 2010/63/EU on the protection of animals used for experimentation. The experiments were carefully designed to reduce the number of animals used, and every effort was made to minimize any suffering or discomfort.

### 2.3. Animal Model of Perinatal Hypoxia-Ischemia

Pregnant Sprague-Dawley rats were acquired at the School of Veterinary Sciences (University of Buenos Aires, Buenos Aires, Argentina) and transported to our vivarium one week before their delivery date. Each dam was housed individually in a maintained 12:12 h light/dark cycle (lights on at 7 a.m.) and a controlled temperature (21 ± 2 °C) and humidity (65 ± 5%) environment. Animals had free access to food (Purina chow) and tap water.

The model used in the present study has been modified from the one developed by Rice et al. [32] and Vanucci et al. [33]. This new version has been previously validated by López-Aguilera et al. [34] and Romero et al. [19]. Postnatal day 7 (P7) male Sprague-Dawley rat pups, obtained from the previously mentioned dams, were anesthetized using 40 mg/kg ketamine and 4 mg/kg xylazine. Animals were placed on a heat plate to keep their body temperature at a constant 37 °C during the procedure. An incision was made on the neck and the right common carotid artery (CCA) was exposed, isolated and permanently ligated with a 6–0 surgical silk thread (carotid (CARO) group *n* = 11). The wound was sutured and pups were returned to their dams for 4–5 h to recover. Afterwards, animals were subjected to a 100% nitrogen environment at 37 °C for 3 min to induce hypoxia. The sham group (SHAM) (*n* = 12) consisted of P7 male rat pups in which their right CCAs were exposed but not ligated, and no hypoxia was performed. Twenty-three days post injury (P30), animals were sacrificed, and their retinas were collected for further analysis.

#### 2.3.1. Tissue Processing

To obtain tissue for Western Blot analysis, six animals per group were euthanized by decapitation; retinas were dissected, homogenized in ice-cold lysis buffer (10 mM Tris/HCl pH 7.4, 10 mM NaCl, 3 mM MgCl2, 0.1% NP-40, protease inhibitors) and sonicated. Samples were centrifuged at 13,000 rpm for 15 min at 4 °C and the supernatants were collected.

For immunohistochemistry, the remaining six animals from each group were anesthetized using 28% (*w/v*) chloral hydrate, 0.1 mL/100 g of body weight. To perform the intracardiac perfusion, freshly prepared 4% paraformaldehyde (Sigma-Aldrich, St. Louis, MO, USA) in 0.1 M phosphate buffer, pH 7.4 was used. The thoracic cavity was opened and a 24 G butterfly needle was carefully inserted in the left ventricle. Saline solution (0.9% sodium chloride), with 1% (*v*/*v*) heparin, was applied at a rate of 9.99 mL/min to clean the tissues. After 5 min of sodium chloride cleansing, a cold 4% formaldehyde solution (freshly made from paraformaldehyde; Sigma-Aldrich, St. Louis, MO, USA) was applied for 20–30 min. When the tissues were properly fixated, retinas were dissected and post-fixed in the same solution for 2 h. Coronal sections (4 μm thick) were cut using a Leica sliding microtome and then recovered for light microscopy studies.

#### 2.3.2. Immunohistochemistry

Immunohistochemistry was carried out as described in Romero et al. [19]. Prior to the staining, paraffin was dissolved, and sections were hydrated and incubated in 3% hydrogen peroxide for 10 min to quench endogenous peroxidases. After three washing steps in PBS, non-specific antibody binding sites were blocked with 10% goat serum (Invitrogen Corporation, Camarillo, CA, USA) diluted in PBS and sections were incubated overnight with primary antibodies (rabbit anti-Nrx (1:1000, 16128-1-AP, Proteintech, Manchester, UK); mouse anti-Nestin (1:200, Millipore MAB353); mouse anti-NF(s) (1:1000, Sigma NO142); goat anti-SOX2 (1:400, Santa Cruz sc17320); mouse anti-iNOS (1:500 BDTransduction Laboratories 610599)) at 4 °C. Sections were washed and subsequently incubated with goat anti-rabbit or anti-mouse fluorescent secondary antibodies (1:500, BA-1000, Vector Laboratories Inc., USA) for 60 min at RT and mounted with Mowiol (Sigma-Aldrich, St. Louis, MO, USA). Sections without primary antibody were used as a control to verify the specificity of the secondary antibody. Samples were examined by fluorescence microscopy using a Leica TCS SP5 microscope with a 63-fold/1.4 oil lens and a 20-fold air lens. Images were analyzed and compiled using Adobe Photoshop 11.0 CS4. Note that for protein staining, all samples (both SHAM and CARO groups) were processed in parallel using the same antibody dilutions. Images were quantified using Image-J software. In order to determine the percentage of reactive area in ex vivo retinas, images were converted to 32-bits and the appropriate threshold was settled for each marker. The whole area corresponding to a retinal layer was delimited and the percentage of reactive area was measured.

### 2.4. Cell Model: Hypoxia-Reoxygenation in ARPE-19 Cells

An established [35], spontaneous cell line from the retina of a young 19-year-old male donor (ARPE-19) was used in the present study. The phenotype of the cells was assessed and verified by real-time-PCR showing that they expressed the RPE cell markers RPE65 and Best1. Cells were thawed and maintained in culture flasks containing 50% HämsF12 medium, 50% DMEM, supplemented with 10% fetal calf serum, 100 U/mL penicillin and 0.1 mg/mL streptomycin. Cells were cultured at 37 °C in a 90% humidified atmosphere containing 20% O_2_ and 5% CO_2_. For hypoxia and reoxygenation, cells were cultured at 1% O_2_ and 5% CO_2_ for 24 h. Afterwards, part of the cells was reoxygenated at 20% O_2_ for another 24 h.

#### 2.4.1. Gene Expression

RNA was extracted from cultured ARPE-19 cells using the GeneJet RNA Purification Kit (Thermo scientific, Waltham, MA, USA) following the manufacturer’s instructions. 1 μg RNA was used for cDNA synthesis (Thermofisher, Waltham, MA, USA) in the Tgradient Thermocycler (Biometra, Göttingen, Germany). cDNA synthesis was carried out for 10 min at 25 °C, followed by a 2 h step at 37 °C and reverse transcriptase inactivation at 85 °C for 5 min. The gene expression of the internal control GAPDH (Fwd: GGATTTGGTCGTATTGGG; Rev: GGAAGATGGTGATGGGATT), NXN (Fwd:GTCATCTCGGATCACCAGC; Rev: GATCGTCTTCGTGTCCTCG or GAACCGAGCAATGGCAGAC and Fwd GAGATCGTCTTCGTGTCCTC; CCTCCTTGATCTTCCGGTAG); SOX2 (Fwd: GGGAAATGGGAGGGGTGCAAAAGAGG; Rev: TTGCGTGAGTGTGGATGGGATTGGTG), Nestin (Fwd: CAGCGTTGGAACAGAGGTTGG; Rev: TGGCACAGGTGTCTCAAGGGTAG) and nNOS (Fwd: TGGCACAGGTGTCTCAAGGGTAG; Rev: GGTTGTCATCCCTCATCCG) were analyzed in duplicates using the AB 7500 Real-Time PCR System (Life Technologies, Waltham, MA, USA). Quantitative real-time PCR was carried out with the following reaction program: 95 °C for 5 min, 45 cycles at 95 °C for 15 s, 60 °C for 20 s and 72 °C for 35 s. To detect potential primer-dimers, an additional dissociation step was performed at 95 °C for 15 min, 60 °C for 1 min and 95 °C for 15 s. Quantitative gene expression was quantified using the comparative threshold cycle method, in seven independent samples. PCR products were analyzed using gel electrophoresis (2% agarose gels).

#### 2.4.2. Western Blotting

Cells were collected and alkylated for 20 min using 100 mM NEM/PBS. Samples were lysed using NEM lysis buffer (40 mM HEPES, 50 mM NaCl, 1 mM EDTA, 1 mM EGTA, pH 7.4, 100 mM NEM, 1% CHAPS, protease and phosphatase inhibitors) for 20 min and were frozen at −80 °C. Samples were thawed on ice and centrifuged at 13,000 rpm for 15 min at 4 °C. Samples were analyzed for total protein concentration using Bradford solution (Biorad, Munich, Germany) in 96-well plates using bovine serum albumin (BSA) as standards. Western blot analysis was performed as previously described in Romero et al. [19]. 10–20 µg of protein were diluted in sample buffer (0.3 M Tris/HCl, pH 7, 50% glycerol, 5% SDS, 1 mM EDTA, 0.1% bromphenol blue), and treated with 100 mM freshly prepared DTT for 30 min at RT followed by 10 min at 94 °C. Samples were then subjected to SDS–PAGE analysis. Membranes were blocked with blocking buffer (5% non-fat milk powder and 1% BSA in Tris-buffered saline with 0.05% Tween-20), incubated with specific primary antibodies at 4 °C overnight and the corresponding secondary HRP-coupled antibody the following day at RT for 1 h. The antigen-antibody complex was developed using an enhanced chemiluminescence method, and luminescence was recorded using a gel documentation system (ChemiDoc™ XRS+ System, Biorad, Munich, Germany). The total amount of protein present in each lane was quantified using the stain-free technology of Biorad and was used for normalization of the blotting data obtained from densitometric analysis [19]. The optical density of each band was determined using ImageJ. The following primary antibodies were used: anti-Nrx (1:1000, 16128-1-AP, Proteintech, Manchester, United Kingdom), anti-GFAP (1:1000, Z0334, Dako, Heidelberg, Germany), anti-MCT4 (1:500, sc-1084, Santa Cruz, CA, USA), anti-NF(s) (1:1000, 2838, Cell Signaling, Danvers, MA, USA), anti-NeuroD1 (1:500, sc-1084, Santa Cruz), β- catenin (Santa Cruz) and p-β-catenin (Millipore). Secondary antibodies used were anti-rabbit (Biorad, Richmond, CA, USA), anti-rabbit (Biorad, Munich, Germany), anti-mouse (Biorad, Munich, Germany) and anti-goat (Biorad, Munich, Germany).

#### 2.4.3. Immunocytochemistry

ARPE-19 cells were plated on glass coverslips covered with 100 µg/mL fibronectin in PBS in 24 well plates. After the induction of hypoxia and reoxygenation, cells were washed with PBS and fixed in 4% paraformaldehyde (Sigma) for 30 min at 4 °C, permeabilized with 0.4% Triton X-100 for 30 min at RT. Samples were blocked using 3% BSA/PBS for 30 min and incubated overnight with the corresponding primary antibody at 4 °C. The following day cells were washed and incubated with anti-mouse IgG secondary antibody (1:400) for 1 h at RT. The actin cytoskeleton was stained using 1:1000 Phalloidin-546 (Invitrogen, Karlsruhe, Germany); nuclei were stained incubating with 1 µg/mL DAPI (Sigma, Roedermark, Germany), in TBS-T solution, for 5 min. Samples mounted using Mowiol mounting medium (Sigma-Aldrich) and examined using a Leica TCS SP5 microscope with a 63-fold/1.4 oil lens and a 20-fold air lens. Images were analyzed using ImageJ (Fiji) and compiled using Adobe Photoshop 11.0 CS4. In order to determine changes in cell size we used Image J. Each cell area was delimited using the freehand line tool and the area of the cell was measured.

#### 2.4.4. Nrx Knock-Down

ARPE-19 cells were transiently transfected with Nrx siRNA (sense: GCC GAU AGC UGA GAA AAU CTT, antisense: GAU UUU CUC AGC UAU CGG CTG). Unspecific control (Scr) siRNA (sense: CAU UCA CUC AGG UCA UCA, antisense: CUG AUG ACC UGA GUG AAU) was used as a control. Three-and-a-half million ARPE-19 cells were resuspended in 550 µL electroporation buffer (21 mM HEPES, 137 mM NaCl, 5 mM KCl, 0.7 mM Na2HPO4, 6 mM D-glucose, pH 7.15), mixed with 15 µg siRNA and were electroporated at 250 V, 1500 µF and 500 Ω using a BTX ECM630 electroporator [9]. FCS was immediately added to the transfected cells, and they were seeded out in 1:5 conditioned medium (1 part old and 4 parts fresh medium). To sufficiently knock-down Nrx, cells were transfected a second time after 3 days.

#### 2.4.5. MTT Assay

To determine cell proliferation/viability a MTT assay was performed according to the manufacturer’s instructions. Cells were seeded out and were cultured in 96 well plates. They were washed once with PBS and treated with 5 mg/mL MTT (3-(4,5-dimethylthiazol-2-yl)-2,5-diphenyltetrazolium bromide) for 30 min at 37 °C. The product of the MTT in living cells was dissolved in DMSO for 30 s and the absorbance was measured at 550 nm.

#### 2.4.6. 2D and 3D Morphology Analysis

For 2D analysis, transfected ARPE-19 cells were seeded in 24 well plates coated with fibronectin. Cells were cultured at 37 °C, 90% humidity and 5% CO2. Two-dimensional cell morphology was analyzed using an inverted cell culture Axiovert 40 CFL microscope (Carl Zeiss, Oberkochen, Germany). Photographs were taken with an Axiovert 40 CFL microscope (Carl Zeiss, Oberkochen, Germany). The cell area was measured using ImageJ, the whole cell contour of 15 cells per well were carefully delimited using the Freehand selection tool and the total area was recorded. For 3D-analysis, transfected ARPE-19 cells were seeded in 24 well plates filled with a collagen matrix. To generate a solid matrix, a collagen layer without cells was set at 37 °C for 4 h. Afterwards, a second layer of collagen with transfected cells was seeded. Liquid medium was added over the second collagen layer to avoid dryness of the matrix. Cells were cultured for 24 h at 37 °C, 90% humidity and 5% CO_2_. Cells were analyzed using an inverted cell culture Axiovert 40 CFL microscope (Carl Zeiss, Oberkochen, Germany). Photographs were taken with an Axiovert 40 CFL microscope (Carl Zeiss, Oberkochen, Germany) and changes in morphology were recorded. Images were analyzed using FIJI-Image J.

#### 2.4.7. Sholl Analysis

Sholl analysis is a quantitative method used to determine the number of projections and the complexity of the ramifications that some cells present. Supplementary Figure S1 depicts a cell studied using Sholl analysis, a central point (red cross) is set in the middle of the cell soma. From this point, several radial circumferences are set (red dotted lines). These circumferences are going to be intersected by different cell projections and their ramifications. In the figure, the intersections for the first circumferences are depicted in green, the following pink, yellow and blue. Note that the more projections and/or ramifications, more intersections with the circumferences are found. Photographs were converted to 8 bits and the threshold was set. The cell soma was selected using the multipoint tool. Six shells with radius from 0 to 25 μm (5 μm apart) were set using the Neuroanatomy-Sholl plugin.

#### 2.4.8. VEGF Release

ARPE19 cells were seeded out in 96 well plates and were incubated as indicated. Release of VEGF was measured in supernatants using the human VEGF-Duo Set ELISA from R&D systems according to the manufacturer’s instructions. Medium was used as blank to verify VEGF from the FCS. To rule out cell lysis or unspecific release, lactate dehydrogenase (LDH) was also measured using the CytoTox 96^®^ Non-Radioactive Cytotoxicity Assay (Promega, Walldorf, Germany). The data were correlated to the amount of viable cells and analyzed by CellTiter-Blue assay (CellTiter-Blue^®^ Cell Viability Assay, Promega, Germany).

### 2.5. Statistical Analysis

Data were examined by *t*-tests and one-way ANOVA tests followed by post-hoc tests corrected by Bonferroni’s correction for multiple comparisons. Results were expressed as mean ± SD. A probability was considered as significant at 5% or less. Statistical analyses were performed using Graphpad Prism 8.

## 3. Results

### 3.1. Nrx Levels Are Increased in Retinas Exposed to Neonatal Hypoxia-Ischemia

**Figure 1:**
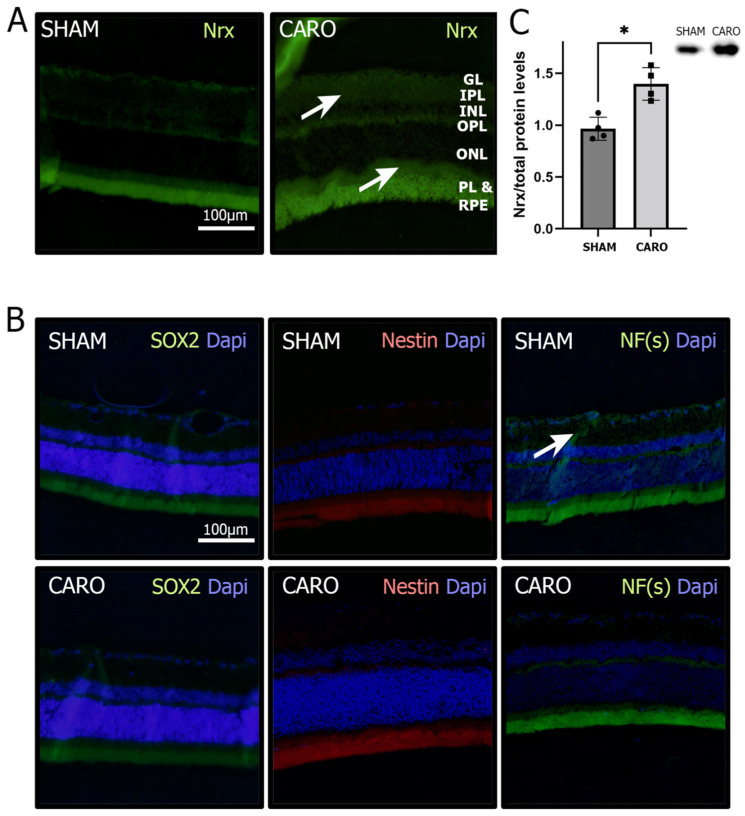
Changes in the retina due to H-I. (**A**). Immunofluorescence staining for Nrx (green) and Dapi counterstaining (blue) in SHAM and CARO retinas. White arrows show increased immunolabeling (**B**). Immunofluorescence staining for differentiation markers: SOX2 (green), Nestin (red), NF(s) (green) and Dapi counterstaining (blue) in SHAM and CARO retinas. White arrow shows increased immunolabeling (**C**). Nrx levels in retinas exposed to H-I (CARO group) and control conditions (SHAM) analyzed using immunofluorescence (Nrx in green) and Western blot (*p* = 0.0286; *n* = 4). Bars and error bars represent the mean + standard deviation (SD) and the asterisk represents a significative difference.

We used Sprague Dawley rats and exposed them to an anoxic-ischemic insult to study Nrx in an ex vivo model for perinatal H-I. We performed Western blot (WB) analysis to determine the protein levels of Nrx in the retinas of 30 days old rats exposed to a neonatal anoxic-ischemic insult (CARO) and control (SHAM) rats. We observed that Nrx levels were significantly higher (37.7%) in the CARO group compared to the SHAM group (*p* < 0.05) (Figure 1C). In order to determine, in which layers of the retina the levels and distribution of Nrx changes, we performed immunofluorescence analysis. We observed that Nrx presents a very faint staining in the plexiform layers, nuclear layers, the photoreceptor layer (PL) and the RPE in the SHAM control samples. Particularly in the ganglion layer (GL), the inner plexiform layer (IPL), the outer plexiform layer (OPL) and the RPE, there was an increase in the antibody staining in the CARO samples compared to the SHAM control (Figure 1A).

To analyze the role of Nrx in the differentiation of cells in the retina, we looked for several differentiation markers in the different layers of the retina using immunofluorescence. We stained for Nestin (expressed in early stages of differentiation), neurofilaments (NFs, neuronal marker) and SOX2 (expressed in non-differentiated cells). The three markers showed positive staining in the PL and RPE in SHAM retinas. Only NF(s) showed positive staining in both plexiform layers in the SHAM group. However, we were able to observe a decrease in NF(s) in the IPL in the CARO group compared to the SHAM group (Figure 1B).

### 3.2. RPE Cells Exposed to Hypoxia and Reoxygenation Present Higher Levels of Nrx Protein but Lower Levels of Nrx mRNA

**Figure 2:**
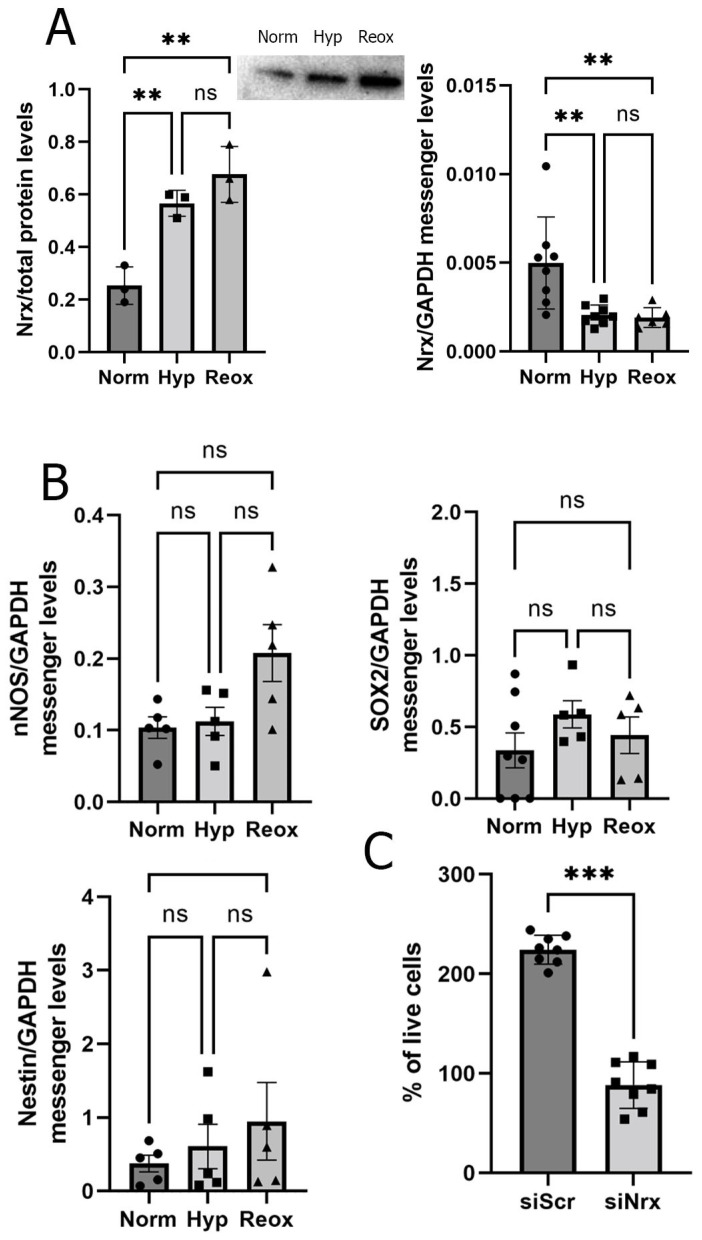
Nrx changes in ARPE-19 cells. (**A**). Nrx protein (*p* = 0.0067, 0.0015; *n* = 3) and mRNA levels in ARPE-19 cells exposed to hypoxia and reoxygenation (*p* = 0.0053, 0.0068; *n* = 6). (**B**). qRT-PCR analysis using differentiation markers (SOX2, Nestin and nNOS) in ARPE-19 cells exposed to hypoxia and reoxygenation (*p* = ns; *n* = 5). (**C**). MTT assay displaying cell proliferation/viability in control ARPE-19 cells (siScr) or cells with transient Nrx knock-down (siNrx) (*p* = 0.0002; *n* = 8). Bars and error bars represent the mean + standard deviation (SD). **, *** Asterisks represent different levels of significance.

Early experiments, carried out in 1980 by Weiter and Zuckerman [36], suggested that the RPE, along with the PL, account for two thirds of the oxygen consumption in the retina. Furthermore, an oxygen decrease in the RPE produces deleterious alterations in the PL [37]. Since the PL and RPE presented changes in Nrx staining in our studies, we decided to focus on analyzing changes related to Nrx levels in an in vitro model using the ARPE-19 cell line. We exposed ARPE-19 cells to three different conditions: 20% oxygen (considered as normoxia) for 24 h, 1% oxygen (hypoxia) for 24 h and 1% oxygen for 24 h, followed by 20% oxygen (reoxygenation) for another 24 h. We analyzed the expression of Nrx via qRT-PCR using three pairs of primers. PCR analysis showed a significant decrease in Nrx mRNA in cells exposed to hypoxia (58.8%) and reoxygenation (61.62%) (Figure 2A). By means of Western blot, we found that Nrx protein levels were significantly higher in cells exposed to hypoxia (92.3%) and reoxygenation (117.5%) when compared to those observed in cells exposed to the normoxic condition (*p* < 0.005) (Figure 2A). To analyze if the increase in Nrx protein levels in ARPE19 cells is associated with changes in differentiation in these cells, we analyzed messenger levels of Nestin, Sox2 and nNOS (necessary for neuronal differentiation; [38]). We did not find significant differences between any of the markers under normoxia, hypoxia and reoxygenation (Figure 2B).

### 3.3. Nrx-Silenced ARPE-19 Cells have Lower Survival Levels and an Altered Morphology

**Figure 3:**
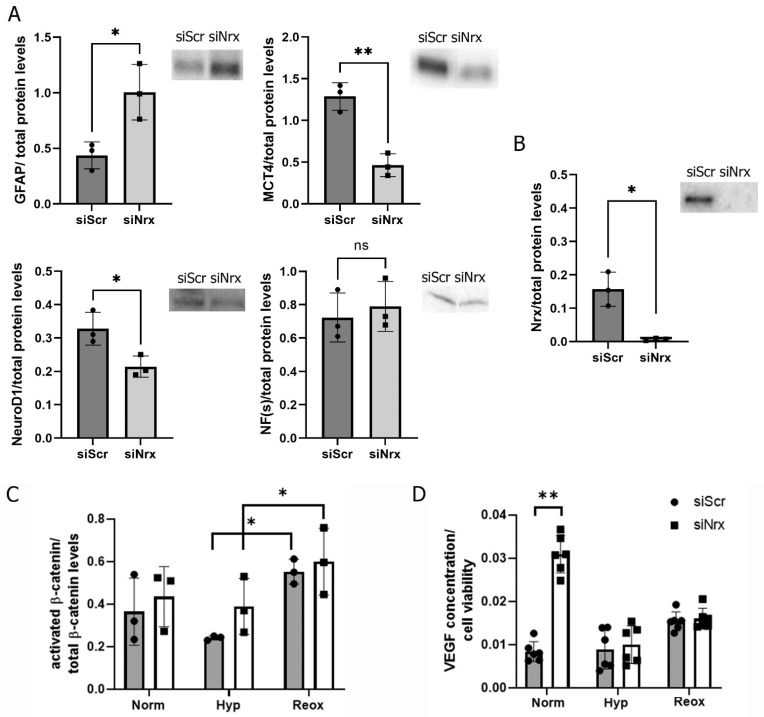
Protein levels in ARPE-19 cells. (**A**). Western blot analysis of differentiation markers: GFAP (*p* = 0.0239; *n* = 3), MCT4 (*p* = 0.0027; *n* = 3), NeuroD1 (*p* = 0.0281; *n* = 3) and NF(s) in siScr and (**B**)**.** siNrx ARPE-19 cells. C. Nrx protein levels analyzed using Western blot (*p* = 0.0361; *n* = 3). (**C**). Western blot analysis of activated β-catenin over total β-catenin levels in siScr and siNrx ARPE-19 cells exposed to H-I and reoxygenation (siScr: *p* = 0.0272; *n* = 3, siNrx: *p* = 0.0133; *n* = 3). (**D**). ELISA assay for VEGF levels in siScr and siNrx ARPE-19 cells (*p* = 0.0063; *n* = 6). *, ** Asterisks represent different levels of significance.

Since we found changes in Nrx levels in retinas exposed to H-I, we propose that these changes can lead to modifications in the retina due to RPE changes, such as dedifferentiation and redifferentiation of this group of cells. To analyze the function of Nrx in these cells, we used the ARPE19 cell line and RNA-interference to silence Nrx expression. The knock-down efficiency was 95.6% compared to control cells that were transfected with unspecific scrambled siRNA, as determined by Western blotting and densitometric analysis using ImageJ (Figure 3C).

**Figure 4:**
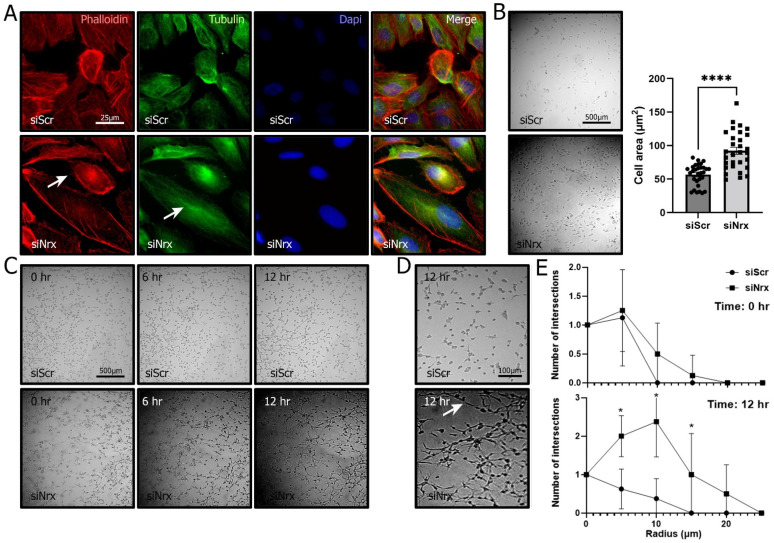
Nrx knock-down affects ARPE19 morphology and number of protrusions. (**A**). Immunofluorescence staining of the cytoskeleton: Actin (phalloidin, red), Tubulin (green) and nuclei counterstaining (Dapi, blue) in siScr and siNrx ARPE-19 cells. (**B**). Light microscopy area analysis of siScr and siNrx ARPE-19 (*p* = < 0.0001; *n* = 40–50). (**C**,**D**). Light microscopy photographs of 3D morphology analysis at different time points of siScr and siNrx ARPE-19 cells. (**E**). Sholl analysis of siScr and siNrx ARPE-19 cells at 0 h and 12 h after plating (*p* = 0.000128, 0.000098, 0.019188; *n* = 20). Bars and error bars represent the mean + standard deviation (SD). *, **** Asterisks indicate different levels of significance.

Firstly, we were interested in analyzing if the lack of Nrx affected cell viability/proliferation. Therefore, we cultured ARPE-19 cells under normoxia, hypoxia and reoxygenation and performed viability assays. We were able to observe a significant decrease in cell viability/proliferation (62.3%; *p* < 0.01) when Nrx was knocked-down in ARPE-19 cells under normoxic conditions (Figure 2C). Next, we analyzed ARPE-19 morphology. Following transient knock-down of Nrx, cells were cultured on fibronectin-coated glass slides. We assessed cell morphology by immunocytochemistry using phalloidin to stain actin and a specific antibody against tubulin. Cells were analyzed by fluorescent microscopy and ImageJ. Cells transfected with the siNrx presented a more elongated phenotype when compared to control cells; the number of F-actin nanotubules was increased and the tubulin staining was more concentrated near the nucleus (Figure 4A). Furthermore, using light microscopy, and a lower magnification, we were able to perform a detailed quantification of ARPE-19 cell morphology in a high number of cells per field; this analysis showed an increase in cell size in cells in which Nrx had been silenced (62.7%; *p* < 0.01) (Figure 4B).

RPE cells are found in a complex 3D milieu that is hardly reflected by cells plated on fibronectin-coated glass slides. Thus, in a second approach, we studied the morphological changes also in a collagen matrix. Control cells presented a sphere-like morphology with no prolongations. Cells lacking Nrx showed a star-like morphology characterized by long projections allowing more cell contacts (Figure 4C,D). Sholl analysis showed a significant increase in the number (2.4-fold; *p* < 0.01) of projections in ARPE-19 cells in which Nrx was knocked down (Figure 2E).

### 3.4. Nrx Silencing Induces Changes in ARPE-19 Cell Differentiation Levels

Previous works have shown that Nrx affects cell differentiation [2]. Our results indicate that Nrx is essential for proliferation and RPE morphology maintenance. Therefore, we hypothesized that the cells lacking Nrx may be differentiating. We examined the expression of different marker proteins that are known to be related to morphological changes and that affect the differentiation state of cells: Glial fibrillary acidic protein (GFAP) is a structural cytoskeletal protein specifically expressed in astrocytes [39] and in neuronal progenitors [40,41], NeuroD1 is a transcriptional activator involved in the formation of early ganglion cells [42], NF(s) are intermediate filaments found in neurons [43]. ARPE-19 cells in which Nrx was silenced, presented significantly higher levels of GFAP (by 31.7%, *p* < 0.05) and lower levels of NeuroD1 (by 40.1%, *p* < 0.05). NF(s) levels were not significantly different between both groups (Figure 3A). Finally, we analyzed the levels of MCT4 (monocarboxylate transporter 4), a protein that is expressed in astroglia during all stages of development (including mature astrocytes) and it is not expressed in neurons [44,45]. This protein is highly expressed in the basolateral face of RPE cells [46] and changes in its expression may indicate changes in cell differentiation. MCT4 levels were significantly lower (by 58.3%; *p* < 0.05) in cells lacking Nrx compared to control cells (Figure 3A).

### 3.5. Nrx May Affect Cell Differentiation via VEGF Rather Than the Wnt/β-Catenin Pathway

The data previously obtained could indicate that ARPE19 cells are differentiating. It was previously described that Nrx blocks the Wnt/β-catenin pathway [6], which is known to be activated by oxidation [4]. Since we observed changes in cell proliferation and differentiation in cells in which Nrx levels significantly decreased, we aimed to determine if this led to changes in the Wnt/β-catenin pathway. Using WB, we were able to determine that the levels of activated non-phosphorylated β-catenin, that can translocate to the nucleus, do not change in ARPE-19 cells in which Nrx is silenced (*p* = ns) compared to the siScr group (Figure 3C), indicating that the changes in survival/proliferation are not related to this pathway. Interestingly we saw an increase in activated β-catenin in ARPE-19 cells exposed to reoxygenation, compared to cells exposed to hypoxia. This change was observed in both groups of cells (siScr and siNrx) (Figure 3C). Another pathway that is linked to redox regulation and hypoxia is the VEGF (vascular endothelial growth factor) pathway. VEGF levels are linked to neuronal differentiation in neuroblastoma [47] and embryonic cells [48,49]. We hypothesized that the lack of Nrx in ARPE-19 cells affects VEGF expression and thereby the differentiation state of the cells. We analyzed released VEGF via a specific ELISA. Our results indicate that VEGF levels in ARPE-19 cells in which Nrx was silenced are significantly increased (72.92%) compared to the control (*p* = 0.0063) (Figure 3D). We did not find any significant differences between the groups in H-I and reoxygenation treatments. However, the increase in VEGF levels observed in cells in which Nrx is silenced decreases when these cells are exposed to H-I and reoxygenation (*p* = 0.0017 and *p* = 0.0016, respectively).

## 4. Discussion

In the present study, we observed that Nrx protein levels increase in retinas of animals exposed to neonatal H-I, indicating that Nrx may play an important role in H-I. Changes in the levels of this redoxin are sustained after 23 days post injury. The increase in Nrx levels after H-I may be due to a system response to the increment of reactive species produced during a hypoxic-ischemic event [50]. Cellular accumulation of reactive species triggers Nrx protection against these molecules through activation of different enzymes related to H_2_O_2_ metabolism [8].

Our results showed that the RPE layer is one of the layers that presents changes regarding Nrx expression due to H-I. It is known that these cells are of upmost importance for the maintenance of the retinal function [23,24]. Thus, we continued analyzing Nrx changes in RPE cells. To further determine the nature of the alterations produced in Nrx expression during neonatal H-I, we analyzed the expression of this redoxin in ARPE-19 cells. We observed that, like in the animal model, Nrx levels were increased due to the hypoxic event and during reoxygenation. However, Nrx mRNA levels are decreased in ARPE-19 cells exposed to H-I, this reduction could indicate that the upregulation of Nrx does not occur at a transcriptional level in H-I but either on a translational level or due to a longer life span of the protein.

Funato et al. [6] demonstrated that Nrx silencing results in an increase in HEK293 cell proliferation. In the present work, we observed a significant reduction in ARPE-19 viability/proliferation when Nrx was silenced. These results are not in accordance with what has been previously found in HEK293 cells. However, while ARPE19 cells present the original human genome [51] and low proliferation rates [52], HEK293 cells present and express E1A/E1B adenoviral proteins that affect cell cycle and counteract apoptosis [53,54]. Furthermore, it has already been proven that the expression and function of Trx proteins is not the same in different cell types [55,56]. Our results indicate that Nrx may either be essential for cell survival, that it is essential to sustain a certain proliferation rhythm or that it is involved in both.

Despite not observing changes in SOX2 or Nestin levels in retinas exposed to H-I, we were also able to observe lower levels of NF(s) in the IPL of retinas exposed to H-I (Figure 1C). This decrease is consistent with the neuronal loss that occurs due to H-I [57]. It is particularly noteworthy that these changes were observed 30 days after birth and 21 days after the hypoxic insult; however, maturation of the retina in rats occurs between 30 and 40 days after birth [58]. Thus, further experiments with longer time points could reveal a reversion of these increased levels, or shed light on whether these changes are permanent in animals exposed to H-I. In line with the evidence obtained from the animal model, we found no changes in SOX2, nNOS or Nestin messenger levels in ARPE-19 cells exposed to hypoxia and reoxygenation. However, we found a significant decrease in VEGF protein levels in ARPE-19 cells exposed to hypoxia and later reoxygenation (Figure 3D).

After determining that Nrx expression is indeed affected by hypoxia and the following reoxygenation, and showing the importance of Nrx in RPE cell viability, we aimed to determine further roles of this redoxin in RPE cells. The morphological analysis showed that ARPE-19 cells are indeed affected by the absence of Nrx. Cells lacking Nrx were bigger overall and presented a more elongated phenotype when compared to the control cells. An increase in size is usually related to senescence in ARPE-19 cells [51] and this could be related to the lower viability observed in our MTT assays. In physiological circumstances, cells exist in a complex 3D matrix. Thus, we analyzed RPE cell morphology in a 3D collagen matrix that allows cells to acquire a more physiological phenotype. ARPE-19 cells that did not express Nrx not only lost the round shape observed in the controls, but they also presented long projections and acquired a star-like shape similar to the one that astrocytes and some types of neurons display. The morphological changes observed in ARPE-19 cells lacking Nrx expression do not match morphological changes observed in apoptotic cells [59,60,61]. Furthermore, immunofluorescence did not show nuclear fragmentation in the siNrx cells and we did not find an increase in apoptotic bodies that can be observed with Dapi staining (Figure 4A) [62]. This change of shape is a clear indicator that the presence of Nrx is essential to maintain the normal RPE phenotype and thereby its function. Since it has been shown that Nrx is essential in differentiation processes [4,5,6] and its interaction with Dlv can be regulated by the presence of ROS [5], a decrease in Nrx levels could be leading to the activation of the Wnt/Beta Cat pathway and thus promoting RPE differentiation into a neuronal phenotype.

Due to the morphological changes observed, we proposed that ARPE-19 cells lacking Nrx could be going through differentiation into either neuronal or glial cell types. To test this hypothesis, we analyzed the levels of different glial and neuronal differentiation markers. We observed a significant increase in GFAP. Since GFAP is a filament that is expressed in astrocytes [39] and neuronal progenitors [40,41], its increase in ARPE-19 cells could be the result of cell differentiation. We could also see a decrease in NeuroD1 levels; this protein is a transcription activator that directs differentiation of RPE cells towards ganglion cells [42]. Regarding NF(s), the most abundant intermediate filament in mature neurons [63], we could not see any changes in its levels. This result and the decrease in NeuroD1 levels could be indicating that RPE cells are not differentiating towards ganglion cells. However, at this stage the increase in GFAP levels could be an indication of an early neuronal differentiation. It was previously shown that GFAP levels increase in cells that are in the early stages of neuronal differentiation [64]. The decrease in MCT4 levels could indicate that these cells are not following the astrocytic pathway either. It is clear, however, that RPE cells lost their epithelial phenotype and that changes, at a molecular level, are taking place. These changes could be important for RPE cells functionality related to differentiation and maintenance of the retinal structure and visual function [23,24].

Nrx blocks the Wnt/β-catening pathway, thus we proposed that silencing Nrx expression in ARPE-19 cells could affect the Wnt/β-catening pathway, activating it and inducing differentiation. However, in the absence of Nrx, there was not a decrease in β-catening phosphorylation in ARPE-19 cells. Other Wnt signaling pathways related to Dvl, such as the Wnt Planar Cell Polarity Pathway (PCP), may also be activated due to the lack of Nrx [65,66]. Indeed, if ARPE-19 cells change morphology into a star-shaped one, the polarization present in this type of cells should be affected [67,68].

Weng et al. [47] demonstrated that VEGF levels are related to neuronal differentiation. Furthermore, it has been shown that VEGF induces neuronal differentiation in embryonic cells [48,49]. In the present study, we found an increase in VEGF levels in ARPE-19 cells subjected to Nrx gene silencing. We propose that this increase could be related to changes in ARPE-19 morphology and a possible cell differentiation of RPE cells into a neuronal phenotype. The activation of the Wnt/β-catenin promotes VEGF transcription [61]. However, VEGF acts as a guiding factor in cell orientation during blood vessel formation [69], linking it to the PCP. Furthermore, VEGF acts as a mitogen not only for neurons but also for glial cells [48] and it has been previously shown that an increase in this growth factor is related to the onset of proliferative diabetic retinopathy [49,70,71,72].

## 5. Conclusions

Changes in Nrx expression in animals subjected to H-I could be leading to alterations in the organization of the retina, due to modifications in RPE survival rates, morphological changes and alterations in differentiation markers. Nrx is essential to maintain cell morphology in vitro, and our results point towards an essential role in maintaining the RPE cell differentiation state. All these regulatory changes are not related to the Wnt/β-catening pathway and seem to be related to VEGF.

## Data Availability

Data is contained within the article and supplementary material.

## Supplementary Materials

The following supporting information can be downloaded at: https://www.mdpi.com/article/10.3390/antiox11061106/s1, Figure S1: (A). Scheme depicting a cell with several projections and Sholl analysis. Central point (red cross) is set in the middle of the cell soma. From this point, several radial circumferences are set (red dot lines). These circumferences are intersected by different cell projections and their ramifications. The intersections for the first circumferences are depicted in green, the following pink, yellow and blue. (B). Graphic showing how the results of this lone cell would look in a graph. Y axis; number of intersections, X axis Circumference number; Figure S2: (**A**). Quantitative analysis of immunofluorescence staining in retinas from SHAM and CTL animals, stained with Nrx and three differentiation markers (SOX2, Nestin and NF). (**B**). Quantitative analysis of cell area of immunofluorescence staining performed in ARPE-19 cells, both in control (siScr) and with Nrx silencing (siNrx).
