# Supplementary material for: Nucleoredoxin Plays a Key Role in the Maintenance of Retinal Pigmented Epithelium Differentiation
Source: Antioxidants (Basel). 2022 Jun 1;11(6):1106. doi: 10.3390/antiox11061106 (PMC9220054; doi:10.3390/antiox11061106)
Supplement: Supplementary file 1 [file antioxidants-11-01106-s001.zip › antioxidants-1715564-supplementary.pdf]

Supplementary Materials

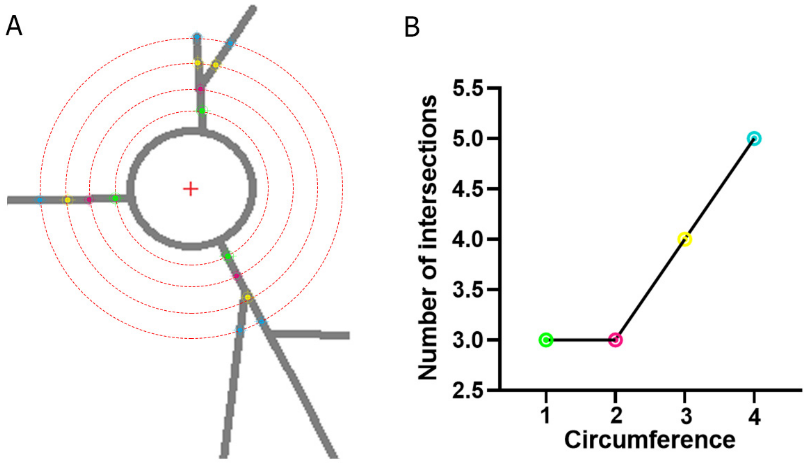

Supplementary Figure S1: A. Scheme depicting a cell with several projections and Sholl analysis. Central point (red cross) is set in the middle of the cell soma. From this point, several radial circumferences are set (red dot lines). These circumferences are intersected by different cell projections and their ramifications. The intersections for the first circumferences are depicted in green, the following pink, yellow and blue. B. Graphic showing how the results of this lone cell would look in a graph. Y axis; number of intersections, X axis Circumference number.

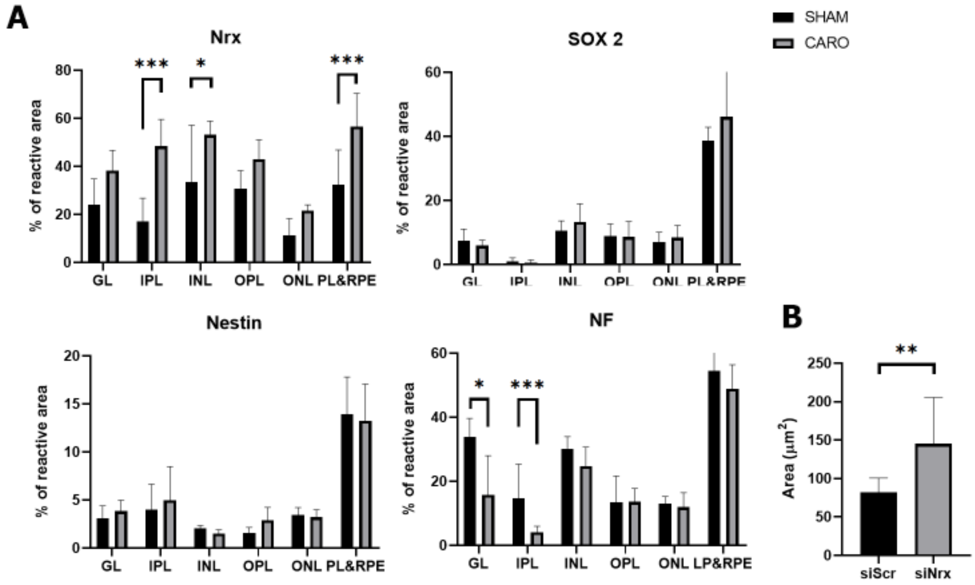

Supplementary Figure S2: A. Quantitative analysis of immunofluorescence staining in retinas from SHAM and CTL animals, stained with Nrx and three differentiation markers (SOX2, Nestin and NF). B. Quantitative analysis of cell area of immunofluorescence staining performed in ARPE-19 cells, both in control (siScr) and with Nrx silencing (siNrx), *, **, *** Asterisks represent different levels of significance.
